# Unnecessary Operations on Women

**Published:** 1906-12

**Authors:** Arthur Dean Bevan

**Affiliations:** Chicago


### CORRESPONDENCE.

### "Unnecessary Operations on Women."

EDITOR SURGERY, GYNECOLOGY AND OBSTETRICS.

SIR: In the last twenty-five years the treatment of disease has become, year by year, more surgical. As a medical student twenty-five years ago, I can remember the limited application of surgery as compared with its range today. In the college clinic we saw fractures and dislocations, stone in the bladder, osteomyelitis, operations for strangulated hernia and extravasted urine amputations, the removal of the breast, an occasional ovariotomy, and operations for malignant tumors. We saw practically nothing of the great mass of work which fills the modern surgical clinic today. No radical cures for hernia, no appendicitis operations, no stomach surgery, no kidney surgery, no gallstone surgery, no hysterectomies, little of modern gynecology, no resections of the intestines for carcinoma, no thyroidectomies, no laryngectomies, no brain or spinal cord surgery. These fields of surgery have practically been created since 1880.

Every one who has lived through the period in question, and who is in a position to compare the work of today with the work of twenty-five years ago, knows that thousands of lives have been saved and thousands of people have been freed from suffering by modern surgery. The advances have been marvelous. Modern surgery has been a great boon to humanity. Modern surgery has been so successful, it has become so easy to secure aseptic results, that there has grown up with it a curse, i. e., the doing of operations which are unnecessary and unwarranted. No class of operators has been entirely free from this curse, but the specialists, with their narrower range of vision and limited work, have undoubtedly suffered more from it than the general surgeon, who, however, has been by no means free from its influence.

The principal sufferers have been women, and the principal offenders have been men who have limited their work to gynecology. Women are the easy victims of the surgeon who advises and performs unnecessary operations. Nine out of ten women who consult a physician because they do not feel well will believe, if they are told so, that their sexual organs are at fault, and will submit to an operation if it is suggested that it is necessary for a cure. The result has been that where the general practice of medicine shows a wider application of surgical therapeutics than formerly, the practice of gynecology has become wholly surgical. There are many gynecologists who do not think of taking charge of a woman except for an operation, and the percentage of women who apply to them for advice, and in whom they find no indication for an operation, is so small as hardly to form, as the chemist would say, a trace in the sum total. This situation is an interesting study, and I shall endeavor to give my impressions of it.

Are all these operations necessary?

In reply to this question, I shall say that my own impression of the surgical work done on women, especially that done by men who limit their work to gynecology, is that certainly thirty per cent. of it is unnecessary and unwarranted. These unnecessary operations are made up largely of the following:

1. Curettings without pathological warrant.
2. Repair of the ordinary torn cervix.
3. Amputation of the cervix.
4. The repair of the relaxed outlet, without any visible impairment in function.
5. The many operations for retroposition of the movable uterus.
6. The operations for so-called cystic degeneration of the ovary,—a condition which is found in almost all female cadavers, and which is physiological and not pathological.
7. The removal of the uterus for small innocuous fibroids.
8. The fixing of the palpable right kidney, which is so common in women, that, depending on the personal equation of the operator, it can be found in from ten per cent. to thirty per cent. of women.
9. Operations such as resection or removal of ovaries because they are believed to be the cause of reflex symptoms in stomach, back, etc.

If I am right in my statement that thirty per cent. of these operations are unnecessary and unwarranted, what is the explanation?

Are these operators dishonest?

Are they ignorant?

Are they misguided surgical enthusiasts?

The answer is, that some of them are dishonest, some are ignorant, and some are misguided surgical enthusiasts.

Some are dishonest, and operate for the patient's fee.

Some are ignorant of broad pathological principles, and operate on these cases because they have been taught by their professors and colleagues to do so.

Some are misguided enthusiasts, who are honest, who have good anatomical and pathological training, but who have so limited their vision to their specialty that they believe that it and they are the center of the pathological female human universe, about which all else revolves.

If I am right in my statements, what is the remedy for this condition, which so menaces modern medicine? I believe that it is publicity; frank, open discussion in just such journals as this, which is not limited to a single speciality, but is devoted to the broad field of obstetrics, gynecology, and surgery.

A discussion in our medical societies of the subject of unnecessary and unwarranted operations might accomplish much good. Medical students must not be trained by the men whose misguided enthusiasm is responsible for most of this work. Operators must be made to realise more fully the great responsibility which is assumed in undertaking any operation, and must be made to see the criminal side to the unnecessary operation. Operators must broaden their horizon to cover the entire human body. The successful surgeon of the future will be the skilled general diagnostician who can operate. The gynecologist of today is extending his field and is operating on the appendix or kidney, gall-bladder or stomach, in many cases which, ten years ago, he would have submitted unsuccessfully to operations on the uterus and ovaries. The wider the territory which he covers, the better gynecologist will he become, and the fewer unnecessary operations will he perform.

I have limited my remarks to unnecessary operations on women because these present such a glaring evil, and I have singled out the gynecologist because he is responsible for much of this work. The same criticisms are to be made of the general surgeon or the operator in any specialty, who for fee, or through ignorance or misguided enthusiasm, submits a human being to the risks and costs of an unnecessary and unwarranted operation. All praise to the splendid achievements of modern surgery, all honor to the modern surgeon who gives his patients the benefit of operations which relieve suffering and prolong life; but what of him into whose hands a patient's life has been intrusted, and who for fee or fame, because of ignorance or enthusiasm, risks this life by an operation which is not necessary and is unwarranted?

We should recognise the existence of this evil and make every effort to do away with it.

Chicago. ARTHUR DEAN BEVAN.

---

